# Genetics Research turns a new [open access] leaf…

**DOI:** 10.1017/S0016672318000071

**Published:** 2019-02-07

**Authors:** Mónica B. J. Moniz, Fiona G. Hutton

**Affiliations:** Cambridge University Press, Cambridge, UK

The journal *Genetics Research* was first launched as *Genetical Research* in 1960 with the goal of publishing “original work in English on all aspects of genetics, or in any field which has an important bearing in genetics”. The Executive Editor was E. C. R. Reeve of the Institute of Animal Genetics, Edinburgh. The journal was well received by the community and received high accolades from the leading microbiologist J. A. B. Stocker (Stocker, 1962) who described the journal as publishing high quality papers that dealt with “mammals (mostly mouse), arthropods (mostly *Drosophila)* and microorganisms”. The journal went on to publish seminal papers including papers on: Inbreeding in artificial selection programs (Robertson, 1961); gene conversion in fungi (Holliday, 1964); linkage and artificial selection (Hill & Robertson, 1966); hitch-hiking effects of favourable genes (Smith & Haigh, 1974); and later analysis of gene expression microarray data (Kerr & Churchill, 2001). By 2007, the journal title was considered outdated; it was felt the original name reflected early research in the field rather than the latest cutting edge topics in genetics, and so the title *Genetics Research* was introduced. In 2013, Noam Shomron, based at Tel Aviv University, was appointed as the new Editor in Chief and the journal focused on publishing research that combined high-throughput techniques, bioinformatics, human genetics and human genetics disease research.

A major goal of Cambridge University Press is to support Open Research, of which Open Access publishing is an essential pillar. As such, the Press has been analysing the needs of the communities that publish in our journals, ascertaining which titles would develop and flourish under the open access model. We know that the scholarly communications landscape is undergoing rapid change, with some funders making Gold OA a requirement. Moreover, an increasing number of institutes in Europe, and a few in the US, are endorsing and promoting Gold OA. This waive of endorsement has culminated on the creation of cOALition S, comprised of a group of eleven European national research funders and the European Commission. cOALition S has announced a new open access policy – Plan S (cOAlition, 2018). Plan S stipulates that by 2020 scientific publications that result from research funded by public grants (provided by participating national and European research councils and funding bodies), must be published in compliant Open Access Journals or on compliant Open Access Platforms.

Mindful of this evolution, we are delighted to announce that from January 2019, *Genetics Research* will convert to the open access model of publication. Genetics is an area where both authors and funders are showing substantial support for Open Access, with increasing author uptake of OA. It is our belief that converting *Genetics Research* to open access is the right solution for the journal and for the community. From January 2019, *Genetics Research* will publish all articles under the Creative Commons Attribution License (CC BY), which permits use, distribution, reproduction and adaptation in any medium, provided the original work is properly cited. Subsequently, an Article Processing Charge (APC) will be payable by authors or their funder on acceptance of their primary research article. In the majority of cases, these costs are paid by the author, his or her institution, or a funder.

From January 2019, we are also introducing a new editorial board with a focus on creating an internationally representative and gender balanced board. We are broadening the scope again respecting the mission of the journal when it was created. We are also making efforts to encourage open research by endorsing strong open data policies.

We hope you are as enthusiastic by these changes as we are and that you contribute by submitting your work, by engaging with the journal content as a reader, and by providing your expert peer review advice, make this journal an important venue for your community. We would like to thank you for your continuing support and endorsement of the journal as we move towards an exciting future.
